# Cystic Fibrosis-Screening Positive Inconclusive Diagnosis: Newborn Screening and Long-Term Follow-Up Permits to Early Identify Patients with CFTR-Related Disorders

**DOI:** 10.3390/diagnostics10080570

**Published:** 2020-08-08

**Authors:** Alice Castaldo, Chiara Cimbalo, Raimondo J. Castaldo, Marcella D’Antonio, Manuela Scorza, Laura Salvadori, Angela Sepe, Valeria Raia, Antonella Tosco

**Affiliations:** 1 Department of Translational Medical Sciences, Cystic Fibrosis Centre, University Federico II, Via Sergio Pansini 5, 80131 Naples, Italy; chiara.cimbalo@unina.it (C.C.); raim.castaldo@studenti.unina.it (R.J.C.); salvadori.laura@libero.it (L.S.); ornellasepe@hotmail.com (A.S.); raia@unina.it (V.R.); antonella.tosco@unina.it (A.T.); 2 CEINGE-Advanced Biotechnology, Via Gaetano Salvatore 486, 80145 Naples, Italy; dantonio@ceinge.unina.it (M.D.); scorza@ceinge.unina.it (M.S.)

**Keywords:** cystic fibrosis, CF-SPID, CFTR-RD, newborn screening, genotype–phenotype correlation

## Abstract

Background: Newborn screening (NBS) early-identifies cystic fibrosis (CF), but in CF-screening positive inconclusive diagnosis (CF-SPID) the results of immunoreactive trypsinogen (IRT), molecular analysis and sweat test (ST) are discordant. A percentage of CF-SPID evolves to CF, but data on long-term monitoring are lacking. We describe the follow-up of all CF and CF-SPID identified between 2008 and 2019. Methods: NBS was performed by IRT followed by molecular analysis and ST between 2008 and 2014; double IRT followed by molecular analysis and ST after 2014. Results: NBS revealed 47 CF and 99 CF-SPID newborn, a ratio 1:2.1—the highest reported so far. This depends on the identification by gene sequencing of the second variant with undefined effect in 40 CF-SPID that otherwise would have been defined as carriers. Clinical complications and pulmonary infections occurred more frequently among CF patients than among CF-SPID. Two CF-SPID cases evolved to CF (at two years), while eight evolved to CFTR-related disorders (CFTR-RD), between one and eight years, with bronchiectasis (two), recurrent pneumonia (four, two with sinonasal complications), recurrent pancreatitis (two). No clinical, biochemical or imaging data predicted the evolution. Conclusion: Gene sequencing within the NBS reveals a higher number of CF-SPID and we first describe an approach to early identify CFTR-RD, with relevant impact on their outcome.

## 1. Introduction

Cystic fibrosis (CF) is a systemic autosomal recessive disorder due to variants in the *cystic fibrosis transmembrane conductance regulator* (*CFTR*) gene that impair the protein activity causing a defective transport of chloride through the respiratory, biliary, gastrointestinal and reproductive epithelia bringing on the secretion of thick mucus [1]. CF is a life-limiting disease, even if the clinical expression and the outcome of the disease are widely heterogeneous even among patients with the same genotype and among affected sib-pairs [2]. Furthermore, the number of patients diagnosed with less severe, usually monosymptomatic disease enclosed under the term of CFTR-related disorders (CFTR-RD) is increasing and although such patients have a better outcome in comparison to patients with CF, they may develop late complications [3,4].

The immediate access to specialized follow-up and cares is the most relevant point in the outcome of patients with CF. For this reason, newborn screening (NBS) programs for CF are available in many countries. The procedures for NBS are widely different between countries and regions, but in most cases they include the analysis of immunoreactive trypsinogen (IRT) on dried blood spots followed by molecular analysis and sweat test (ST).

Ideally, patients with CF would show a concordance of altered IRT, two *CFTR* causing-disease variants and altered ST (i.e., sweat chloride > 60 mmol/L), while healthy subjects would result negative to all the three approaches. Really, it is emerging a growing number of cases in which the screening parameters are not concordant, also as most of the 2000 *CFTR* variants described so far in patients with CF or CFTR-RD [5] have unclear or variable phenotypic consequences and so ST may result intermediate (i.e., between 30 and 59 mmol/L).

In particular, there are infants with NBS screen positive, but (i) none or 1 CF variant and intermediate ST (i.e., 30–59 mmol/L); (ii) 2 *CFTR* variants, one or both with unclear clinical consequences, with ST < 30 mmol/L. Such cases were defined as CFTR-related metabolic syndrome (CRMS) [6] or CF screen positive with inconclusive diagnosis (CF-SPID) [7]. The number of patients with CF-SPID varies, depending on the approach used for NBS [8]. Furthermore, most infants with CF-SPID are asymptomatic at the newborn period; they may evolve to CF with a different frequency and time. It was suggested the follow-up of infants with CF-SPID for three years after birth, but no data are available for longer outcomes [8].

The NBS for CF in Campania Region (Southern Italy, about five million of inhabitants) started on all newborns in 2016 after a previous pilot period between 2008 and 2015. The aim of the present study is to describe the procedures of CF-SPID identification, including results that we obtained from the follow-up of all CF-SPID and CF patients in the last eleven years.

## 2. Materials and Methods

This study is part of a larger research study on CF-SPID, coordinated by the regional CF center of Tuscany (Italy) that involves several Italian regions. The study was approved by the Ethical Committee of the coordinator center, approved code 140/2018, approved on 1 October 2018 (i.e., Giannina Gaslini Institute, Florence, Italy) and by the Ethical Committee of the University of Naples Federico II.

We collected the data of all infants diagnosed as CF or CF-SPID by NBS from 2008 to 2019 in Campania Region, Southern Italy. We identified 47 patients with CF and 99 with CF-SPID. These subjects were monitored in our center in the last eleven years.

### 2.1. The Protocol of NBS

During the pilot phase (2008–2013) we performed: (i) a first IRT on dried blood spot collected in 3rd day; we referred to a cutoff value of 48 ng/mL; (ii) a second IRT in 20th day of life in all infants positive to the first IRT; (iii) molecular analysis independently by the result of the II IRT; (iv) gene sequencing, ST and a clinical evaluation in all cases with one or two variants at first panel. Since 2015, our protocol of NBS for CF includes: (i) a first IRT performed on dried blood spot collected in 3rd day; we referred to a cutoff value of 48 ng/mL as suggested by the SIMMESN (i.e., the Italian Society for Metabolic Diseases and Neonatal Screening); (ii) a second IRT in 20th day of life in all infants positive to the first IRT; for the second IRT we used a cutoff value of 37 ng/mL; (iii) molecular analysis, ST and a clinical evaluation in all cases positive to the second IRT and in all cases that at the first IRT had a value > 100 ng/mL. The *CFTR* genotype was defined by the first level molecular analysis testing for 40 most frequent variants and for variants peculiar to Southern Italy. Thus, we performed the search for large gene deletions [9] and gene sequencing [10] in all cases that had: (i) a *CFTR* variant at the first level analysis; (ii) sweat chloride levels > 30 ng/mL. To define the effect of *CFTR* variants we referred to the *CFTR*2 and to the French *CFTR* databases [11,12]. Segregation analysis testing intragenic *CFTR* gene repeats was performed in families that asked for carrier or prenatal diagnosis, in which the proband had one or both unknown mutations [13].

### 2.2. The Follow-up of Infants with CF-SPID

All the infants with CF were diagnosed according to defined criteria [14]. We diagnosed as CF-SPID all the infants that had the first (2008–2013) and the second IRT (after 2014) positive and: (i) none or 1 *CFTR* variant associated with intermediate sweat chloride (i.e., 30–59 mmol/L); or (ii) 2 *CFTR* variants, one or both with unclear clinical consequences, associated with a ST < 30 mmol/L).

All infants with CF-SPID were monitored every six months with a protocol that included clinical, laboratory and imaging evaluation. IRT was analyzed by immunofluorescent assay (GPS neonatal IRT, GPS instrument). Sweat test was analyzed according to guidelines always in the same laboratory under interlaboratory quality control programs [15]. The pancreatic evaluation was performed testing fecal pancreatic elastase-1 (using a cutoff of 200 mcg/g) measured in the absence of acute pancreatitis or gastrointestinal diseases; pancreatitis was defined as acute or chronic according to the report from International study group of pediatric pancreatitis [16] excluding all known causes of pancreatitis. The evaluation of pulmonary airway infection/colonization was evaluated by sputum (in greater children) or oropharyngeal swab (in younger patients) culture. Chronic infection was defined according to the modified Leeds criteria [17]; furthermore, in addition to the clinical evaluation of pulmonary disease, we performed TC scan if necessary. The evaluation of the liver status and the presence of CF liver disease (CFLD) were evaluated on the basis of clinical, biochemical and U/S scanning (if necessary) results. The evaluation of sinonasal pathology was performed by clinical evaluation followed by rhinoscopy and, if necessary, by TC scanning, as described [18]. Furthermore, we recorded other complications, i.e., meconium ileus, metabolic alkalosis and distal intestine obstructive syndrome (DIOS) as described [2]. Finally, we recorded all biochemical parameters [19], the number of hospital access and therapies [20].

## 3. Results

**Figure 1:**
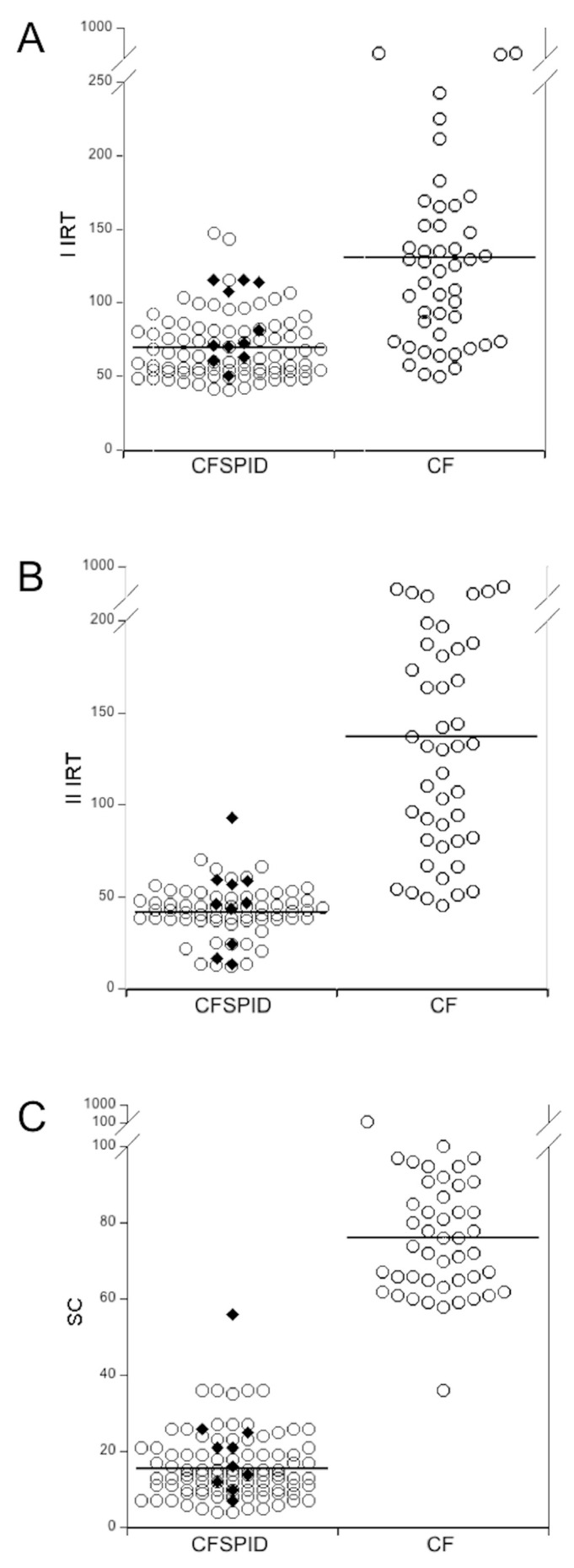
(**A**) Scattergram of the first immunoreactive trypsinogen (IRT), ng/mL. (**B**) second IRT, ng/mL and (**C**) and sweat chloride (SC), mmol/L in patients with CF and in subjects with CF-SPID at the newborn period. Among the subjects with CF-screening positive inconclusive diagnosis (CF-SPID), the black diamonds represent the cases evolved to cystic fibrosis transmembrane conductance regulator (CFTR)-related disorders (CFTR-RD) or to CF.

Between 2008 and 2019 the NBS program revealed 47 patients affected by CF (21 males, 44.7%) and 99 newborns with CF-SPID (53 males, 53.5%). All the 47 patients with CF had two positive IRT; 43/47 (91.5%) had sweat chloride > 60 mmol/L, while four of them had intermediate results (36, 58, 58 and 59 mmol/L, respectively). Furthermore, all the 47 CF patients had two *CFTR* variants (Supplemental Table S1); in 44/47 (93.6%) cases, both the variants were disease-causing according to *CFTR*2 and to *CFTR*-France databases [11], while in 3/47 cases one variant was disease-causing and the other was not reported in the databases, i.e., H147P (1 case) and 1249-1G>A (2 cases). All these three patients had two pathologic sweat test. Table 1 reports the results of the first and of the second IRT performed at the newborn period in 47 CF patients and in 99 subjects with CF-SPID and the results of sweat chloride. All the three parameters resulted significantly higher in patients with CF than those with CF-SPID (see also Figure 1).

Among the 99 subjects with CF-SPID, 5 had sweat chloride between 30 and 59 mmol/L and one or two *CFTR* variants with unclear phenotypic consequences. While, 93/99 cases diagnosed as CF-SPID had sweat chloride < 30 mmol/L and two *CFTR* variants of which 1 or none known as CF causing, according to the *CFTR*2 database (Supplemental Table S2).

After the NBS and the counseling of the parents, 46/47 (97.9%) patients with CF were included in our follow-up program, while 1/47 is now under follow-up in another CF regional center. Among the 99 newborns with CF-SPID, 80 (80.8%) were included in our follow-up program, while 5/99 are now under follow-up in other CF regional centers and 14/99 (14.1%) were lost to follow-up. Table 2 shows: (i) the number of newborn tested for NBS; (ii) the number of cases positive to the I IRT; (iii) the number of cases positive to the II IRT; (iv) the period of follow-up for all the subjects currently under study: 32/46 (69.6%) patients with CF are followed since at least three years, while among CF-SPID subjects, 64/80 (80%) are under follow-up since at least three years.

Table 3 shows a comparison of respiratory infections between the patients with CF and the subjects with CF-SPID, recorded during the follow-up. The occurrence of acute infection by *p. aeruginosa* is significantly more frequent among patients with CF and the same is true for acute episodes by *S. maltophilia*. Acute infections by methicillin-sensitive *S. aureus* were more frequent among subjects with CF-SPID, while colonization by such strain resulted more frequent in patients with CF. All respiratory infections either acute or chronic by other bacteria had the same recurrence between the two groups of subjects.

Table 4 reports the comparison of clinical complications between CF and CF-SPID subjects. Meconium ileus, pancreatic insufficiency, CF-liver disease, single episodes of pneumonia as like as recurrent pneumonia bronchiectasis, atelectasis, metabolic alkalosis and dehydration resulted significantly more frequent among patients with CF than among CF-SPID subjects. Interestingly, some complications as recurrent pancreatitis, recurrent pneumonia, bronchiectasis, chronic sinusitis and nasal polyps justified diagnosis as CFTR-RD in patients previously recorded as CF-SPID.

Table 5 reports the data of NBS obtained in the 2 subjects with CF-SPID evolved to CF and in the 8 evolved to CFTR-RD. The data of the I and the II IRT of subjects evolved, were not significantly different than the whole group of CF-SPID subjects (see Figure 1). Similarly, the ST was < 30 mmol/L in one of the two CF-SPID evolved to CF and intermediate in another, while it was < 30 in all CF-SPID evolved to CFTR-RD. Table 5 reports also the data of ST at the time of the final diagnosis of CF or CFTR-RD. Both the cases evolved to CF had two altered ST while among the cases evolved to CFTR-RD 7/8 cases had sweat chloride < 30 mmol/L also at the final diagnosis, and only 1 patient had the intermediate value of 35 mmol/L of sweat chloride. Both cases evolved to CF at 2 years of age while it ranged between 1 and 8 years old in cases evolved to CFTR-RD, with 4/8 cases in which the final diagnosis of CFTR-RD was performed > 3 years old. Table 6 reports the clinical evolution of subjects with CF-SPID. The two patients evolved to CF received the final diagnosis at two years for the presence of two consecutively positive ST. Their characteristics are summarized in Table 5 and Table 6. Both of them had pancreatic sufficiency and resulted free from typical CF clinical complications so far. Furthermore, none of them experienced pulmonary colonization by the typical CF pathogens; only one of the two patients was colonized by *K. oxytoca* from the age of 1 year. Table 6 also shows the clinical data of CF-SPID subjects evolved to CFTR-RD that permitted the final diagnosis: two cases had bronchiectasis; 4 cases had recurrent pneumonia (two of which also with sinonasal complications); two cases had recurrent pancreatitis. Four out of eight patients had experienced *P. aeruginosa* respiratory infections, in two cases recurrent.

## 4. Discussion

Our data indicate that: (i) the number of CF-SPID revealed at the NBS is about two-fold the number of patients with CF; (ii) 2 of the 80 (2.5%) subjects with CF-SPID evolved to CF, while eight (10%) evolved to CFTR-RD so far; (iii) half of patients evolved to CFTR-RD were diagnosed > 3 years; (iv) no data at the NBS or during the follow-up was predictive of the evolution from CF-SPID to CF or to CFTR-RD.

The ratio of CF-SPID to CF diagnosed at NBS in the present study (approximately 2:1) is higher than previous studies that reported a ratio between 1:1.5 and 1:6.3 [6,7,8,21]. This depends on: (i) a lower than usual IRT cut off value (i.e., 48 ng/mL rather than the usual 60 ng/mL for the initial IRT and 37 ng/mL for the second specimen); (ii) the cut off values used for sweat chloride; and (iii) the mounting evidence that many more infants than generally accepted who have a high IRT level have CFTR dysfunction. In addition, it may depend also on the use of sequencing analysis of *CFTR* gene performed in all subjects positive to the IRT who carried a variant identified by first level analysis. In fact, after the first level analysis, only 52 of the 92 subjects had the criteria to be defined as CF-SPID, while 40 had a ST < 30 mmol/L and a single variant and therefore they would have been diagnosed as CF carriers. The subsequent gene sequencing revealed a second variant with variable phenotypic consequences, allowing to classify them as CF-SPID. These data are in agreement with the results obtained in California [8], where the use of gene sequencing in the NBS program allowed the identification of a greater number of CRMS infants (CF-SPID) than in other countries. Similarly, four of the eight patients that we diagnosed as CF-SPID at the newborn period who evolved into CFTR-RD (# 1, 3, 4, 6 in Table 5) also had a ST < 30 mmol/L, a single variant after first level analysis, among which the D1152H in four cases [22] and the second variant with undefined phenotypic consequences, i.e., Q1476X, L997F (2 cases) and S1426 F, were revealed by sequencing. Therefore, gene sequencing, which today can be performed with costs comparable to those of commercial panels of variants [10], significantly increases the number of CF-SPID diagnosed, and also other studies suggest performing extended gene analysis in subjects with CF-SPID [8]. Consequently, it allows start a follow-up of such subjects aimed to early reveal cases that may evolve into CF or CFTR-RD.

Furthermore, during the pilot period of our NBS (i.e., 2008–2014), we performed molecular analysis in all subjects positive to the first IRT, independently by the result of the second IRT, followed by sequencing and ST in cases with one identified variant. This permitted to identify as CF-SPID 14 subjects with the second IRT < 37 ng/mL, among which four cases that evolved to CFTR-RD. Thus, the NBS protocol with a single IRT followed by molecular analysis and ST identifies a higher number of CF-SPID than the protocol based on two IRT followed by molecular analysis and ST.

In the present study, only two of our 80 CF-SPID patients (i.e., 2.5%) evolved to CF, differently by other studies that report 3% to 20% of CF-SPID evolved to CF during a three-years follow-up [8,23,24]. This may depend in part by the use of gene sequencing during NBS, considering that also among our patients diagnosed as CF, four cases had an intermediate ST and the second causing variant was identified by the sequencing, allowing to classify such cases as CF and not as CF-SPID. Meanwhile, eight (10%) patients with CF-SPID evolved to CFTR-RD and such figure could be higher, considering that a half of cases were diagnosed as CFTR-RD between four and eight years. In fact, although in our patients with CF-SPID the occurrence of clinical complications and bacterial colonization during the follow-up was significantly more rare than patients with CF, in agreement with previous studies [8,25], overall, subjects with CF-SPID showed a higher frequency of *P. aeruginosa* (29.1%) and *S. maltophilia* (12.7%) infection than the rate < 3.6% reported in healthy children [21]. This suggests that a percentage of such CF-SPID subjects would evolve later. The evolution of 4/8 CF-SPID subjects to CFTR-RD after the third year of life, suggests that the follow-up of CF-SPID must be longer than the two or three years as recently suggested [26] considered that CFTR-RD may develop symptoms later than CF, that most of them are negative to the NBS and that however, the early diagnosis may have a great impact on their final outcome [3,27]. At the state of the art, the identification of CF-SPID patients and their monitoring seems the lone approach to early identify patients with CFTR-RD, and this is the first study that reveals the strategy of the long-term follow-up of CF-SPID subjects to early identify patients with CFTR-RD. Of course, in this approach it is critical to counsel the parents of the infants with CF-SPID to mitigate the psychological impact of the long term follow-up (also because most of them would not evolve to CF or to CFTR-RD) and to avoid the loss to follow-up of their children [8,28,29]. We carefully counseled the parents [6] both at diagnosis and during each visit, and this allowed to reduce the loss of follow-up to less than 15% of cases. However, a careful balance should be performed between the potential advantages of the early diagnosis of CF or CFTR-RD in a percentage of CF-SPID patients, and the costs of the long-term follow-up of all cases of CF-SPID revealed at the NBS.

Furthermore, our data indicate that no biochemical or clinical marker predicts the evolution of CF-SPID to CF or to CFTR-RD. In fact, NBS parameters (i.e., I and II IRT and ST) in the patients evolved to CF or to CFTR-RD were not significantly different than the whole group of CF-SPID subjects, in agreement with the results of Ooi [23,29]. Similarly, the ST at final diagnosis was still < 30 mmol/L in 7/8 of our CF-SPID patients evolved to CFTR-RD and apart from the evidence of the clinical complication that permitted the final diagnosis of CFTR-RD none other clinical or laboratory marker predicted the evolution. Similarly, the *CFTR* genotype of the evolved patients was not particularly suggestive of the clinical course, i.e., four of the eight patients evolved to CFTR-RD had a severe variant *in trans* with the D1152H variant, that has a very heterogeneous clinical impact [22], while both the cases of CF-SPID evolved to CF had a severe variant *in trans* with the [5 T;TG12] complex allele, confirming in turn the heterogeneous clinical impact of such complex allele that was found, *in trans* with a severe variant either in asymptomatic subjects, in CFTR-RD and in CF patients [5]. Furthermore, recently emerged that also CF carriers have a high risk to evolve in a series of CF-related conditions [30]. This suggests that other potential markers would be explored to identify CF-SPID patients with a higher risk to evolve into CF or CFTR-RD and that would require a closer surveillance, like genetic modifiers [31], nasal potentials [4] or the analysis of CFTR protein activity on ex-vivo cells [32].

To conclude: gene sequencing during NBS and the protocol based on the I IRT-molecular analysis-ST led to identify a high number of cases with CF-SPID, 10% of which evolved to CFTR-RD, while only two cases evolved to CF so far. The evolution to CFTR-RD occurred in a range of age between one and eight years, suggesting that the follow-up of subjects with CF-SPID should be higher than three years suggested so far, and this seems the lone approach to early identify patients with CFTR-RD. A careful counseling of the families is necessary to reduce the number of cases lost to follow-up. No clinical, biochemical or imaging data predicted the subjects with CF-SPID at a higher risk of clinical evolution, suggesting the search of other predictive markers. Finally, it would be necessary to define, also through functional studies, the effect of several variants, like the L997F that are classified differently by the different databases.

## Figures and Tables

**Table 1:** Newborn screening of cystic fibrosis (CF) and CF-screening positive inconclusive diagnosis (CF-SPID) subjects.

	*n*	Males	I IRT, ng/mL	II IRT, ng/mL	Sweat Chloride at Diagnosis	In Follow-up
		(*n* and%)	mean and (SD)	mean and (SD)	mmol/L, mean and (SD)	
CF	47	21 (44.7%)	136.8 (72.7)	130.7 (68.2)	76.1 (16.1)	46 * (97.8%)
*p*			<0.001	<0.001	<0.001	
CF-SPID	99	53 (53.5%)	71.53 (22.3)	42.2 (14.6)	16.2 (8.7)	80 ** (80.8%)

* 1/47 patients is now under follow-up in other regional center; ** 5/99 subjects are now under follow-up in other regional center, 14/99 subjects are lost to follow-up. IRT—immunoreactive trypsinogen.

**Table 2:** Newborn screening (NBS) data (2008–2019).

Years of	Year of	Screened	Positive to	Positive to	CF	CF-SPID	CF	CF-SPID
follow-up	NBS	newborn (N)	I IRT (N and%)	II IRT (N and%)	(N and%)	(N and%)	in follow-up	in follow-up
1	2019	46,000	378 (0.82)	60 (0.13)	7 (0.014)	8 (0.017)	7	8
2	2018	48,000	367 (0.76)	63 (0.14)	7 (0.016)	9 (0.020)	7	8
3	2017	50,000	468 (0.94)	59 (0.14)	7 (0.016)	10 (0.023)	7	8
4	2016	50,000	435 (0.87)	61 (0.13)	9 (0.020)	19 (0.041)	9	17
5	2015	21,000	177 (0.84)	45 (0.21)	4 (0.019)	10 (0.048)	3	7
6	2014	28,000	156 (0.56)	47 (0.17)	7 (0.025)	12 (0.043)	7	8
7	2013	17,000	133 (0.78)	32 (0.19)	3 (0.018)	14 (0.082)	3	9
8	2012	11,000	113 (1.03)	28 (0.26)	2 (0.018)	6 (0.055)	2	5
9	2011	7000	78 (1.14)	20 (0.29)	0	1 (0.014)	0	1
10	2010	3500	56 (1.60)	10 (0.29)	1 (0.029)	4 (0.011)	1	3
11	2009	3000	58 (1.93)	8 (0.27)	0	3 (0.011)	0	3
12	2008	3000	65 (2.17)	7 (0.23)	0	3 (0.011)	0	3

CF—cystic fibrosis; CF-SPID—CF-screening positive inconclusive diagnosis; IRT—immunoreactive trypsinogen.

**Table 3:** Respiratory infections in CF and in CF-SPID patients.

	Cystic Fibrosis	*p*	CF-SPID
	(*n* = 46)		(*n* = 80)
*P. aeruginosa* (acute)	23 (50.0%)	0.017	23 (28.7%)
*P. aeruginosa* (colonization)	1 (2.2%)	n.s.	0
MSSA (acute)	13 (28.3%)	0.0036	44 (55.0%)
MSSA (colonization)	26 (56.5%)	<0.001	13 (16.2%)
MRSA (acute)	7 (15.2%)	n.s.	8 (10.0%)
MRSA (colonization)	1 (2.2%)	n.s.	2 (2.5%)
*H. influenzae* (acute)	16 (34.8%)	n.s.	32 (40.0%)
*H. influenzae* (colonization)	2 (4.3%)	n.s.	2 (2.5%)
*S. maltophilia* (acute)	18 (39.1%)	<0.001	10 (12.5%)
*A. fumigatus* (acute)	1 (2.2%)	n.s.	0
*Mycobacteria*	0		0
Other colonization	0		0

CF-SPID—CF-screening positive inconclusive diagnosis; MSSA—methicillin sensitive *S. aureus*. MRSA—methicillin-resistant *S. aureus*.

**Table 4:** Clinical complications in CF and in CF-SPID patients.

	Cystic Fibrosis	*p*	CF-SPID
	(*n* = 46)		(*n* = 80)
Meconium ileus	6 (13.0%)	<0.001	0
Pancreatic insufficiency	39 (84.8%)	<0.001	0
Recurrent pancreatitis	1 (2.2%)	n.s.	2 (2.5%) (2 cases evolved to CFTR-RD)
CF liver disease	5 (10.9%)	0.003	0
CF related diabetes	0	n.s.	0
Acute pneumonitis	19 (41.3%)	<0.001	12 (15.0%)
Recurrent pneumonitis	8 (17.4%)	0.022	4 (5.0%) (4 cases evolved to CFTR-RD)
Bronchiectasis	13 (28.3%)	<0.001	3 (3.7%) (2 cases evolved to CFTR-RD)
Atelectasis	8 (17.4%)	<0.001	0
Chronic sinusitis	2 (4.3%)	n.s.	1 (1.2%) (1 case evolved to CFTR-RD)
Nasal polyposis	2 (4.3%)	n.s.	1 (1.2%) (1 case evolved to CFTR-RD)
Metabolic alkalosis	6 (13.0%)	<0.001	0
DIOS	2 (4.3%)	n.s.	0
Dehydration	5 (10.9%)	0.014	1 (1.2%)

CF-SPID—CF-screening positive inconclusive diagnosis; CFTR-RD—CFTR-related disorders; DIOS—distal intestine obstructive syndrome.

**Table 5:** Data of newborn screening of 8 subjects with CF-SPID evolved to CF (cases 1 and 2) or to CFTR-RD (cases 3 to 10).

#	Sex	Current Age	Final Diagnosis	I IRT	II IRT	SC at Birth	SC at Final Diagnosis	*CFTR* Genotype
		years	and age (years)	ng/mL	ng/mL	mmol/L	mmol/L	
1	F	3	CF, 2	115	47.0	56	87, 84	Y849X//[5T;TG12]
2	M	2	CF, 2	82.1	44.0	7	61, 76	D1152H//[5T;TG12]
3	M	11	CFTR-RD, 8	70.7	16.7	21	28	F508del/Q1476X
4	M	11	CFTR-RD, 8	71.8	13.2	26	27	1717-1G>A/[5T;TG12]
5	M	11	CFTR-RD, 7	61.6	24.1	16	11	F508del/L997F
6	F	7	CFTR-RD, 2	50.8	25	25	35	R334Q/L997F
7	M	6	CFTR-RD, 4	72.9	56.8	14	26	F508del/D1152H
8	F	5	CFTR-RD, 3	63.5	58.4	10	12	S1426F/D1152H
9	M	7	CFTR-RD, 2	108	59.4	21	27	L732X/D1152H
10	M	4	CFTR-RD, 1	116.4	92.8	12	28	F508del/D1152H

CF-SPID—CF-screening positive inconclusive diagnosis; CFTR-RD—CFTR-related disorders; IRT—immunoreactive trypsinogen; SC—sweat chloride.

**Table 6:** Clinical data of 8 subjects with CF-SPID evolved to CF (cases 1 and 2) or to CFTR-RD (cases 3 to10).

#	Pancreatic	Pancreatitis	*P. aeruginosa*	Other	Sinonasal	CFRD	CFLD	Pulmonary Disease
	status			colonization	status			
1	PS	no	no	*K. oxytoca*	no	no	no	no
2	PS	no	no	no	no	no	no	no
3	PS	no	acute, 8 yrs	no	no	no	no	bronchiectasis
4	PS	no	recurrent, 3 yrs	no	no	no	no	bronchiectasis
5	PS	no	recurrent, 1 yr	MSSA	CS, 7 yrs	no	no	recurrent pneumonitis, 7 yrs
6	PS	no	no	no	NP, 3 yrs	no	no	recurrent pneumonitis, 2 yrs
7	PS	no	no	no	no	no	no	recurrent pneumonitis, 1 yr
8	PS	no	no	MSSA	no	no	no	recurrent pneumonitis, 1 yr
9	PS	RP	no	no	no	no	no	no
10	PS	RP	acute, 1 yr	no	no	no	no	pneumonitis, 1 yr

CFRD—CF-related diabetes; CFLD—CF liver disease; PS—pancreatic sufficiency; MSSA—methicillin-sensitive *S. aureus*; CS—chronic sinusitis; NP—nasal polyposis; RP—recurrent pancreatitis. The age is referred to the first episode.

## Supplementary Materials

The following are available online at https://www.mdpi.com/2075-4418/10/8/570/s1, Table S1: *CFTR* genotype of 47 patients with CF, Table S2: *CFTR* genotype of 99 subjects with CF-SPID.

## Non-Standard Abbreviations

CF	cystic fibrosis
CFTR	CF transmembrane conductance regulator
CFTR-RD	CFTR-related disorder
NBS	newborn screening
IRT	immunoreactive trypsinogen
ST	sweat test
CRMS	CFTR-related metabolic syndrome
CF-SPID	CF screen positive with inconclusive diagnosis
IRT	immunoreactive trypsinogen
CFLD	CF liver disease
DIOS	distal intestine obstructive disease
