# Supplementary material for: Cystic Fibrosis-Screening Positive Inconclusive Diagnosis: Newborn Screening and Long-Term Follow-Up Permits to Early Identify Patients with CFTR-Related Disorders
Source: Diagnostics (Basel). 2020 Aug 8;10(8):570. doi: 10.3390/diagnostics10080570 (PMC7460402; doi:10.3390/diagnostics10080570)
Supplement: Supplementary file 1 [file diagnostics-10-00570-s001.zip › supple/Supplemental Table 1.pdf]

Supplemental Table 1: *CFTR* genotype of 47 patients with CF				
HGVS nomenclature		Legacy name		N
c.1521_1523del	c.1521_1523del	F508del	F508del	19
c.1521_1523del	c.3846G>A	F508del	W1282X	3
c.1521_1523del	c.2657+5G>A	F508del	2789+5G>A	2
c.1521_1523del	c.3884_3885insT	F508del	4016insT	2
c.1521_1523del	c.3731G>A	F508del	G1244E	2
c.1521_1523del	c.3909C>G	F508del	N1303K	2
c.1521_1523del	c.1585-1G>A	F508del	1717-1G>A	1
c.1521_1523del	c.2051_2052delAAinsG	F508del	2183 AA>G	1
c.1521_1523del	c.579+1G>T	F508del	711+1G>T	1
c.1521_1523del	c.(3963+1_3964-1)_(*1_?)del	F508del	CFTRdele22-24	1
c.1521_1523del	c.377G>A	F508del	G126D	1
c.1521_1523del	c.1624G>T	F508del	G542X	1
c.1521_1523del	c.1040G>A	F508del	R347H	1
c.1521_1523del	c.1646G>A	F508del	S549N	1
c.1521_1523del	c.1055G>A	F508del	R352Q	1
c.1117-1G>A	c.3964-78_4242+577del	1249-1G>A	CFTRdele22,23	1
c.1585-1G>A	c.1001G>T	1717-1G>A	R334L	1
c.2051_2052delAAinsG	c.1117-1G>A	2183AA>G	1249-1G>A	1
c.3884_3885insT	c.1624G>T	4016insT	G542X	1
c.(53+1_54-1)_(164+1_165-1)del	c.(53+1_54-1)_(164+1_165-1)del	dele2	dele2	1
c.1624G>T	c.440A>C	G542X	H147P	1
c.3472C>T	c.3964-78_4242+577del	R1158X	CFTRdel22,23	1
c.1657C>T	c.579+1G>T	R553X	711+1G>T	1
