# Supplementary material for: Cystic Fibrosis-Screening Positive Inconclusive Diagnosis: Newborn Screening and Long-Term Follow-Up Permits to Early Identify Patients with CFTR-Related Disorders
Source: Diagnostics (Basel). 2020 Aug 8;10(8):570. doi: 10.3390/diagnostics10080570 (PMC7460402; doi:10.3390/diagnostics10080570)
Supplement: Supplementary file 1 [file diagnostics-10-00570-s001.zip › supple/Supplemental Table 2.pdf]

Supplemental Table 2: *CFTR* genotype of 99 subjects with CF-SPID				
HGVS nomenclature		Legacy name		N
c.1521_1523del	c.1210-33_1210-6GT[12]T[4]	F508del	5T;TG12	5
c.1521_1523del	c.3454G>C	F508del	D1152H	5
c.1521_1523del	c.2991G>C	F508del	L997F	5
c.1210-33_1210-6GT[12]T[4]	c.1210-33_1210-6GT[12]T[4]	5T;TG12	5T;TG12	5
c.2991G>C	c.1210-33_1210-6GT[12]T[4]	L997F	5T;TG12	5
c.1210-33_1210-6GT[12]T[4]		5T;TG12		4
c.3731G>A	c.1210-33_1210-6GT[12]T[4]	G1244E	5T;TG12	2
c.3454G>C	c.1210-33_1210-6GT[12]T[4]	D1152H	5T;TG12	2
c.2991G>C	c.1210-33_1210-6GT[12]T[4]	L997F	5T;TG12	2
c.2657+5G>A	c.2991G>C	2789+5G>A	L997F	2
c.3846G>A	c.1210-33_1210-6GT[12]T[4]	W1282X	5T;TG12	1
c.4277C>T	c.3454G>C	S1426F	D1152H	1
[c.220C>T;c.3808G>A]	c.1210-33_1210-6GT[12]T[4]	[R74W;D1270N]	5T;TG12	1
c.1657C>G	c.2815C>G	R553G	H939D	1
c.1040G>A	c.1046C>T	R347H	A349V	1
c.1001G>A	c.2991G>C	R334Q	L997F	1
c.1000C>T	c.350G>A	R334W	R117H	1
c.3209G>A	c.3454G>C	R1070Q	D1152H	1
c.3909C>G	c.1210-33_1210-6GT[12]T[4]	N1303K	5T;TG12	1
c.3909C>G	c.3025G>A	N1303K	A1009T	1
c.3909C>G	c.3154T>G	N1303K	F1052V	1
c.2991G>C	c.1210-33_1210-6GT[12]T[4]	L997F	5T;TG12	1

c.2991G>C	c.3454G>C	L997F	D1152H	1
c.254G>A	c.4242+13A>G	G85E	c.4242+13A>G	1
c.3731G>A	c.1523T>G	G1244E	F508C	1
c.3389G>C	c.1210-33_1210-6GT[12]T[4]	G1130A	5T;TG12	1
c.1521_1523del	c.3454G>C	F508del	D1152H	1
c.1521_1523del	[c.220C>T;c.3808G>A]	F508del	[R74W;D1270N]	1
c.1521_1523del	[c.220C>T;c.601G>A;c.3808G>A]	F508del	[R74W;V201M;D1270N]	1
c.1521_1523del	c.2930C>T;c.1210-33_1210-6GT[12]T[4]	F508del	[S977F;5T;TG12]	1
c.1521_1523del	c.3154T>G	F508del	F1052V	1
c.1521_1523del	c.3205G>A	F508del	G1069R	1
c.1521_1523del	c.2108A>G	F508del	N703S	1
c.1521_1523del		F508del		1
c.1521_1523del	c.1495C>G	F508del	P499A	1
c.1521_1523del	c.14C>T	F508del	P5L	1
c.1521_1523del	c.4054C>G	F508del	Q1352E	1
c.1521_1523del	c.4426C>T	F508del	Q1476X	1
c.1521_1523del	c.3485G>T	F508del	R1162L	1
c.1521_1523del		F508del		1
c.1521_1523del	c.3705T>G	F508del	S1235R	1
c.1521_1523del	c.527G>C	F508del	S176T	1
c.1521_1523del	c.53A>G	F508del	S18G	1
c.1521_1523del	c.325T>C	F508del	Y109H	1
c.3454G>C	c.1210-33_1210-6GT[12]T[4]	D1152H	5T;TG12	1
c.3454G>C	c.1046C>T	D1152H	A349V	1
c.2195T>G	c.3454G>C	L732X	D1152H	1

c.3454G>C	c.2991G>C	D1152H	L997F	1
c.3454G>C	c.65C>T	D1152H	P22L	1
c.3454G>C	c.1040G>A	D1152H	R347H	1
c.2547C>A	c.3454G>C	Y849X	D1152H	1
[c.1727G>C; c.2002C>T]	c.1210-33_1210-6GT[12]T[4]	[G576A;R668C]	5T;TG12	1
c.2657+5G>A	c.1210-33_1210-6GT[12]T[4]	2789+5G>A	5T;TG12	1
c.1210-33_1210-6GT[12]T[4]	c.1210-33_1210-6GT[12]T[4]	5T;TG12	5T;TG12	1
c.377G>A	c.1210-33_1210-6GT[12]T[4]	G126D	5T;TG12	1
c.2547C>A	c.1210-33_1210-6GT[12]T[4]	Y849X	5T;TG12	1
[c.220C>T;c.3808G>A]	c.1210-33_1210-6GT[12]T[4]	[R74W;D1270N]	5T;TG12	1
2620-15C>G	c.1210-33_1210-6GT[12]T[4]	2620-15C>G	5T;TG12	1
c.1210-33_1210-6GT[12]T[4]	c.1523T>G	5T;TG12	F508C	1
c.1210-33_1210-6GT[12]T[4]	c.419C>G	5T;TG12	P140R	1
c.1210-33_1210-6GT[12]T[4]	c.3038C>T	5T;TG12	P1013L	1
c.1210-33_1210-6GT[12]T[4]	[c.1516A>G; c.3503A>G]	5T;TG12	[I506V;D1168G]	1
c.1210-33_1210-6GT[12]T[4]	c.2900T>C	5T;TG12	L967S	1
c.1210-33_1210-6GT[12]T[4]	c.2991G>C	5T;TG12	L997F	1
c.3884_3885insT	c.1210-33_1210-6GT[12]T[4]	4016insT	5T;TG12	1
c.3718-2477C>T	c.1507A>G	3849+10kbC>T	K503E	1
c.3140-26A>G	c.2991G>C	3272-26A>G	L997F	1
c.2657+5G>A	c.1210-33_1210-6GT[12]T[4]	2789+5G>A	5T;TG12	1
c.2657+5G>A	c.1210-33_1210-6GT[13]T[4]	2789+5G>A	5T;TG13	1
c.1585-1G>A	c.1210-33_1210-6GT[12]T[4]	1717-1G>A	5T;TG12	1
c.1585-1G>A	c.601G>A	1717-1G>A	V201M	1
[c.3209G>A; c.1210-33_1210-6GT[11]T[7]]	c.3854C>T	[R1070Q;8T;TG11]	A1285V	1
